# Reply to “Comment on the Correlation between Complete Blood Count Parameters and Appendix Diameter for the Diagnosis of Acute Appendicitis”

**DOI:** 10.3390/healthcare8040462

**Published:** 2020-11-05

**Authors:** Emin Daldal, Hasan Dagmura

**Affiliations:** 1 General Surgery, Gaziosmanpasa University, Kaleardı Mahallesi, Tokat 60250, Turkey; emin.daldal@gop.edu.tr; 2 General Surgery and Surgical Oncology Department, Gaziosmanpasa University, Kaleardı Mahallesi, Tokat 60250, Turkey

## Answer to the Comments

We would like to thank Akbulut and his colleague for reading our article with interest, and for their valuable comments and criticisms. Firstly, they claimed that there was a significant error in grouping patients, in that lymphoid hyperplasia and fecalith were the two most common causes of acute appendicitis, and in that the presence of lymphoid hyperplasia showed histological presence of acute appendicitis. Here, we think that the authors overlooked that lymphoid hyperplasia, which is one of the causes of acute appendicitis, when reported as lymphoid hyperplasia in histopathological examination has different meanings.

The classical hypothesis in the etiology of acute appendicitis is that the occlusion of the appendix lumen with fecalith or lymphoid hyperplasia, causing an increase in intraluminal pressure, causes ischemia in the appendix wall and then necrosis and perforation. However, this hypothesis does not explain all cases of appendicitis. A careful review of the pathological series suggests that few cases have luminal obstruction. Fecalith is found in only 3.6 to 27% of cases of acute appendicitis, and lymphoid hyperplasia is more common in normal appendix than acute appendicitis.

An alternative hypothesis for the etiology of appendicitis is based on the concept that either bacterial or viral enteric infection leads to mucosal ulceration of the appendix and subsequent bacterial invasion by the normal colonic microbiota. The finding that up to 75% of cases of appendicitis demonstrate well-defined superficial mucosal ulceration supports this theory. Furthermore, mucosal ulceration is a more consistent finding than is dilatation of the appendix or the presence of fecalith and is found earlier in the course of appendicitis [1]. As can be seen from here, the etiology of acute appendicitis has not been fully explained yet. Even the role of lymphoid hyperplasia in the etiology of acute appendicitis is still controversial. Histological findings in acute appendicitis vary from minimal focal inflammation to complete necrosis of the appendix wall. In early lesions, neutrophils appear at the base of the crypts, often adjacent to a small defect in the epithelium. After this inflammatory process reaches the submucosa, it quickly spreads to the appendix. For microscopic diagnosis of acute appendicitis, inflammation should extend to muscularis propria [1,2]. Therefore, lymphoid hyperplasia without inflammation is accepted as NORMAL findings.

A new systematic review and meta-analysis studies from the database of 25 trials and 57,357 patients’ appendectomy specimens were evaluated (SURGERY, year 2020) (Goldblum JR et al. Table 2 see below); inflammation-free lymphoid hyperplasia and inflammation-free fecalith were classified as the NORMAL appendix group [3]. In our study, we defined lymphoid hyperplasia patients without inflammation in the histopathological examination as a normal appendix, and hence defined them as negative appendectomy. Interestingly, in a study conducted by Akbulut, we read that he did not even consider 18 lymphoid hyperplasia patients with inflammatory findings as acute appendicitis and rather classified them as an unusual histopathological findings group [4]. In many studies, the result of lymphoid hyperplasia without inflammation was evaluated as a normal appendix [5,6,7,8]. In another study conducted by pathologist Türkcü et al., lymphoid hyperplasia was found to be the most common cause of negative appendectomy [9]. In a study by Akbulut et al., who suggested that we should evaluate lymphoid hyperplasia as acute appendicitis, we believe that their suggestion would somehow bring contradiction; in fact, in their own study, they did not evaluate patients with lymphoid hyperplasia as acute appendicitis as a result of histopathological examination [10]. As a result, there is no error in the grouping of patients and it is correct to evaluate the lymphoid hyperplasia group as a negative appendectomy.


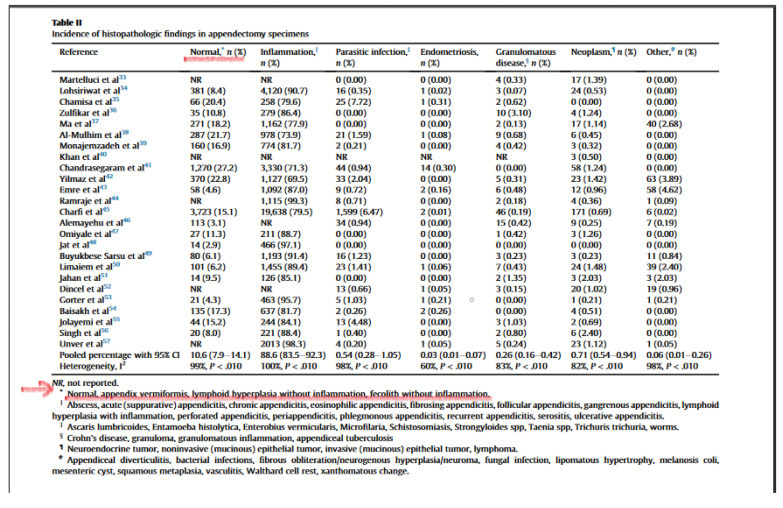


## Answer to Third and Fourth Paragraph

If the “*p*” value found as a result of the correlation analysis is greater than 0.05 (*p* > 0.05), it shows that there is no significant correlation, but if *p* < 0.05, it is statistically significant. If the correlation is statistically significant (*p* < 0.05), the correlation level is interpreted according to the Spearman rho coefficient [11,12]. All correlations in our study are presented in Table 1 in accordance with statistical reporting criteria (APA). In addition, in our study, the correlation between diameter and WBC, lymphocyte, neutrophil, RDW, NLR, and PLT/L was stated to be a weak correlation [2] in the “RESULTS” section. Despite this, the statements we wrote were repeated exactly in the letter to the editor. In other words, they (Akbulut and his colleague) just replicated the results we stated in the article and not the contrary. In addition, they (Akbulut and his colleague) commented on Figure 1 presented in the article only according to their own opinions and thoughts. In Figure 1, the data distribution between the diameter of the appendix and NLR and PLT/L parameters is presented with a scatter plot. The statistical relationship levels between such data are already presented in Table 1.

## Answer to the Fourth Paragraph

In Table 2, the rates of pathology results by diameter groups were compared with the chi-square test and a significant difference was found. They shared additional statistical results using table data.

## Answer to the Fifth Paragraph

We believe that there are no errors in the statistical method or in the title of Table 5. The statements written by Akbulut and his colleague are based on their misunderstanding of their own comments. The statistical method is generally written. Whether some CBC parameters or their ratios are diagnostic and prognostic markers in the diagnosis of appendicitis was investigated by the ROC curve and explicitly written in the statistical method. In the table, the diameter is specified in particular. It will be easily understood by the readers that the CBC parameters referred to in the method are WBC, N, PDW, and NLR. Sensitivity specificity, PPV, NPV, and likelihood ratio (L+) values were calculated and presented in Table 5 using the cut-off values calculated for these four parameters, which are significant in ROC analysis. ROC analysis is needed for the purpose stated in method [3] and the findings are written in accordance with the reporting criteria.

## Answer to Sixth Paragraph

The title of Table 3 is misspelled, but since it is specified as “pathology groups” in the table, it will be understood by the readers that the groups are pathology groups. The weak and strong aspects of imaging methods in diagnosing acute appendicitis are not the purpose of our study. Although there may be a difference between imaging methods in diagnosis, in our study, appendix diameter measurements do not cause heterogeneity, since the diameter is given numerically in both USG and CT.

## Answer to Seventh Paragraph

Akbulut and his colleague made this opinion according to the result of the correlation analysis. In correlation analysis, the diameter was performed using all data without grouping. Statistical results should be evaluated together. When Tables 2–5 are evaluated together, it will be seen that there are no errors in the comments made in the Discussion section. For example, for WBC and N, which are statistically different in Tables 3 and 4, the AUC value in Table 5 greater than 0.70 will indicate that they may be used as a marker for appendicitis discrimination.

In conclusion, as we stated in our article, we think that evaluating the appendix diameter and complete blood count parameters together can be used to increase the diagnostic value and for this purpose, further prospective studies which could be planned in this line of enquiry should include clinical outcome data for sets of patients in which the usefulness of the clinical decision-making utility of these methods is thus evaluated.
